# Discordance between physician-rated health and an objective health measure among institutionalized older people

**DOI:** 10.1186/s12877-015-0074-4

**Published:** 2015-07-09

**Authors:** Javier Damián, Roberto Pastor-Barriuso, Emiliana Valderrama-Gama, Jesús de Pedro-Cuesta

**Affiliations:** National Center for Epidemiology, Carlos III Institute of Health, Av/ Monforte de Lemos 5, 28029 Madrid, Spain; Consortium for Biomedical Research in Neurodegenerative Diseases (Centro de Investigación Biomédica en Red sobre Enfermedades Neurodegenerativas - CIBERNED), Madrid, Spain; Consortium for Biomedical Research in Epidemiology and Public Health (CIBER en Epidemiología y Salud Pública - CIBERESP), Madrid, Spain; “Arroyo de la Media Legua” Primary Care Center, Madrid Health Service (Servicio Madrileño de Salud), Madrid, Spain

**Keywords:** Health assessment, Quality of care, Nursing homes, Mortality, Older people

## Abstract

**Background:**

Although physician-rated health is emerging as a potentially useful variable in research, with implications in practice, it has not been analyzed. Moreover, one of its most important aspects, namely, concordance with patients’ objective health state, has not been investigated. This study sought to measure concordance between physician-rated health and an objective health measure, and assess both measures’ validity in predicting death.

**Methods:**

The data for the study were drawn from a 1998–1999 survey and subsequent mortality follow-up of residential and nursing homes in Madrid (Spain). Study subjects were 630 residents aged ≥65 years, and their respective facility physicians. Measures included agreement between physicians’ rating of residents’ overall health (good, intermediate or poor) and an objective measure of residents’ health (good, intermediate or poor), based on functional capacity, cognitive status, and number of chronic conditions. Overrating was defined as any case where health, rated as good by a physician, was objectively rated as poor.

**Results:**

Whereas 45 % of physicians and 55 % of residents rated their health as good, only 4 % of such residents had good objective health. Of those who received a physician rating of good/very good health, 39.0 % had poor objective health. There was evidence of clear overrating in 18 % of the population, and clear to moderate overrating in 73 % of the population. In terms of power to predict mortality, the pattern of behavior shown by the objective health measure was good, graded and congruent, and better than that shown by physician-rated health.

**Conclusion:**

Physician overrating of the overall health of older persons in residential and nursing homes, would appear to be very high. Although some degree of contextualization by physicians in this setting might be considered reasonable, the degree of overrating in our population seems nevertheless excessive.

## Background

Self-rated health (ascertained via the question, “How would you describe your health: very good, good, fair, poor or very poor?”, or variations of this), is a variable that is widely used and extensively studied, since it provides an easy-to-obtain measure of subjective health, shown to be of great value by innumerable papers [1]. Physician-rated health is a similar measure, which is less used and far less studied and can constitute a very good proxy of health status, a mid-point between self-perceived health and completely objective standards. Physician-rated health has been studied as a comparison measure for self-rated health. For example, Kivinen *et al.* found a weak correlation between older men’s self-rated health and their general health status as evaluated by physicians [2]. Geest *et al.* found discordance between physician- and self-evaluated health in 32 % of cases in a middle-aged primary care population [3]. The predictive properties of physician-rated health have been recently assessed. Among subjects aged 20 years and older, DeSalvo *et al.* found increased mortality in those cases where self-assessed health was worse than physician-assessed health [4]. Giltay *et al.* found that both physician- and self-rated health independently predicted all-cause mortality, albeit with some differences, *i.e.*, while poor self-rated health was associated with cancer mortality, poor physician-rated health was associated with cardiovascular mortality [5]. In terms of mortality, Todd and Goldman reported that interviewer ratings displayed a better predictive behavior than did physician ratings in the case of older adults in Taiwan [6]. Physician-rated health has also been used in a number of studies as an adjustment variable, in an attempt to control for “objective” health status: for instance, Wilper *et al.* observed a higher mortality risk associated with lack of health insurance, controlling for physician-rated health, among other variables [7]; and Moor *et al.* used physician-rated health as an adjustment variable in a study on the association of neuroticism and subjective health [8].

While self-rated health has an inherent value because it is a measure of subjective health, it is highly advisable that physician-rated health be checked against objective measures. Undoubtedly, physicians also incorporate some legitimate subjectivity and may appreciate subtle aspects not captured by objective indicators, yet their assessment is expected to be based on objective health factors to a greater extent than that of other actors, such as nurses, staff, caregivers, the residents themselves, and family members. In principle, congruence between physicians and their patients’ objective situation tends to be taken for granted and, to our knowledge, has not been systematically explored.

It is possible that a marked degree of discordance –particularly in the form of overrating- between patients’ objective health status and physicians’ perception of this could well lead to deficits in preventive interventions, diagnosis, prescribing, treatment and rehabilitation options, and ultimately to an unsatisfactory quality of care. Yet the opposite can also be true, *e.g.*, by limiting iatrogenic actions. The first step however is to examine and explore the nature of physician rating. We were unable to find previous studies that compared physician-rated health against an objective measure of overall health. Consequently, our principal aim was to measure the concordance between physician-rated health and an objective health measure and, in particular, to provide evidence of a specific aspect of discordance, namely, the overrating of health in an institutionalized older population. Our study was essentially descriptive, and rather than seeking to test a specific hypothesis, sought instead to measure and describe potential discordance. In addition, criterion validity (*i.e.*, the measure of how well one variable or set of variables predicts an outcome) for the key variables (physician-rated health, objective health and its components) could be established on the basis of its association with all-cause mortality.

## Methods

### Baseline sampling

Data were obtained from a survey conducted from June 1998 through June 1999, using a probabilistic sample of residents aged 65 years and older drawn from 25 public/subsidized and 30 private residential and nursing homes in Madrid (Spain). Study participants were selected by stratified cluster sampling. Of an initial sample of 800 subjects, 715 responded (overall response rate of 89 %). Due to refusal, prolonged absence or sampling-frame errors, 39 subjects were randomly replaced by a resident of the same facility and sex, yielding a total of 754 interviews.

### Baseline data-collection and variable definition

Using structured questionnaires, data were collected by purpose-trained geriatricians or residents in geriatrics, during interviews conducted with participants, their main caregivers, and facility physicians. Informed consent was obtained verbally from study subjects or their next of kin. The Research Committee of the Carlos III Institute of Health approved the study: this met Spanish legal requirements because at that time there was no statutory need for an ethics committee report in the case of non-experimental research. Patient anonymity was assured through anonymization of the data set. The Carlos III Institutional Review Board also approved the study.

#### Self- and physician-rated health

Subjects were asked about their health via the question, “In general terms, how would you describe your health: very good; good; fair; poor; or very poor?” *Physicians* were asked to rate residents’ health in a similar fashion: “What, in your opinion, is the resident’s health status: very good; good; fair; poor; or very poor?” We deliberately gave no instructions to subjects or physicians in this regard. Interviews with physicians were conducted with the aid of medical records and nursing annotations. For both variables we collapsed the extreme categories to create the following three-category version: good (the result of very good and good); fair; and poor (the result of poor and very poor).

#### Medical conditions

Physicians were asked whether any resident had suffered from one or more of a list of diseases (cancer, obstructive pulmonary disease, arrhythmias, hypertension, ischemic heart disease, congestive heart failure, peripheral arterial disease, stroke in the past year, diabetes, anemia, Alzheimer’s disease, other dementias, Parkinson’s disease, epilepsy, depression, anxiety disorder, arthritis or severe osteoarthritis), and the number of diseases was then computed.

#### Functional status

We used the Barthel Index, as modified by Shah *et al.* [9]. Subjects (55 %) or their main caregivers (45 %) were asked as to residents’ degree of dependence in performing basic activities of daily living (ADL). The following three functional dependency categories were drawn up for the Barthel Index [9]: independent (100 points); mild-to-moderate dependency (61–99 points); severe and total dependency (0–60 points).

*Cognitive status* was evaluated using both the Short Portable Mental Status Questionnaire (SPMSQ, range 0–10 errors) [10], which was suitably amended to adapt to the institutional setting and administered to residents, and the Minimum Data Set Cognition Scale (MDS-COGS, 0–10 point scale) [11, 12], which obtains an assessment from the main caregiver based on selected Minimum Data Set questions. On the basis of these two scale scores, residents were then classified into one of three categories, namely: non-impaired (≤2 education-adjusted SPMSQ errors and ≤1 MDS-COGS points); mild-to-moderate cognitive impairment (3–7 SPMSQ errors and ≤8 MDS-COGS points, or ≤7 SPMSQ errors and 2–8 MDS-COGS points); or severe cognitive impairment (≥8 SPMSQ errors or ≥9 MDS-COGS points). We used this approach because the SPMSQ was not administered to 39 % of individuals for logistic reasons.

We created a single, ordinal, composite index of *objective health*, based on the 3-category version of the following variables: functional status in basic ADLs (Barthel index, three categories); number of chronic conditions (0–1; 2–3; ≥4); and cognitive status (non-impaired; mild-to-moderate impairment; and severe impairment). Subjects in the lowest severity level of the three variables were assigned to “good” health; those in the highest severity level of any of the three variables were assigned to “poor” health; and the remainder were assigned to “intermediate” health state. We applied this scheme in order to be congruent with the notion of these health dimensions being regarded as very important. If a person were to be classified in the severe category of any very important health dimension, no label other than “poor health” would be expected: and, following this same line of reasoning, if someone were to depart from the lowest level of severity in any of these very important dimensions, then such a person should not be assigned a “good health” label. It should be stressed here that only aspects of objective health are being considered. It is perfectly feasible for a person to be in an intermediate or poor (objective) category and yet have a good self-rating, with positive coping abilities and satisfactory social life.

Clear *overrating* was defined as good physician-rated health in cases of poor objective health; and moderate overrating was defined as fair physician rating in cases of poor objective health, or good physician rating in cases of intermediate objective health.

### Mortality ascertainment during follow-up

Study participants were followed up for mortality through September 15, 2013. Mortality was ascertained by mailing a survey to the participating facilities seeking data on residents’ vital status and through linkage to the Spanish National Death Index, which includes all deaths registered in Spain since 1987 [13]. For the present study, residents contributed follow-up time from their 1998–1999 baseline interview until death, age 105 years, or September 15, 2013, whichever occurred first.

### Analysis

Weighted (quadratic weights) kappa coefficients were computed to assess agreement between pairs of the 3-category versions of self-rated health, physician-rated health, and objective health.

In order to explore the association between the different components of the objective health measure and the physicians’ ratings, we fitted a multinomial logistic regression model with physician-rated health as the 3-category dependent variable. Six independent variables were included in this model: baseline age; sex; type of facility; and the 3 components of objective health (functional dependency, number of medical conditions, and cognitive status), entered in a continuous form with values 1, 2, or 3 for the low, intermediate and high severity levels, respectively.

Mortality for the different variables was studied using Cox regression models. We estimated hazard ratios, adjusted for baseline age, sex and type of facility. Non-parametric survival curves were obtained as the baseline survival functions from health-stratified Cox models (good, fair, or poor health) with years from the baseline interview as the time scale. Models were adjusted to the overall weighted percentages of baseline category indicators, including age (65–74, 75–84, or ≥ 85 years), sex (women or men), and type of facility (public, subsidized, or private). Due to the complex sampling and different selection probabilities of the study participants (with residents of public facilities and men being oversampled), all analyses took into account the effect of stratification and clustering on estimates, and were weighted to reestablish proportionality. All analyses were performed using the Stata 13 statistical software package [14].

## Results

**Fig. 1:**
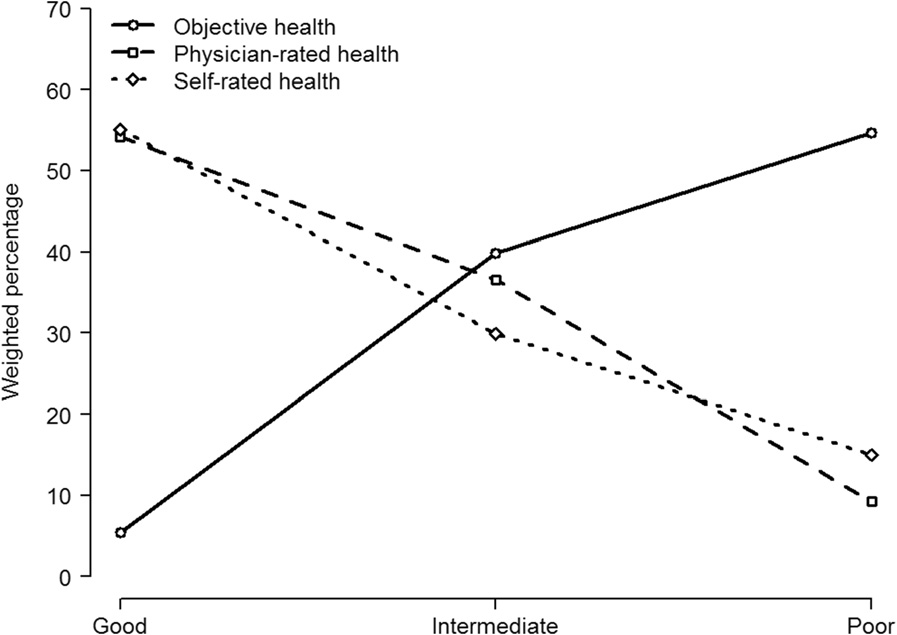
Distributions of objective health, physician-rated health, and self-rated health among Madrid nursing-home residents with no missing values in self-rated health: Spain, 1998–1999. *Good* corresponds to good objective health, good or very good physician-rated health, and good or very good self-rated health. *Intermediate* corresponds to intermediate objective health, fair physician-rated health, and fair self-rated health. *Poor* corresponds to poor objective health, poor or very poor physician-rated health, and poor or very poor self-rated health

Among the 754 subjects studied, 660 had a physician rating, and valid objective health values could be created for 630. Table 1 shows the general characteristics of the participants: in general the profile was typical of an older, institutionalized population. The majority of residents were from large-public or small-private facilities, and the fractions enjoying functional independence (21.7 %) and no cognitive impairment (50.0 %) were fairly high. Table 2 shows the distributions of physician-rated health and objective health. The marginal distributions can be better appraised in Fig. 1, in which the distribution of self-rated health is included. While physician- and self-rated health distributions were very similar, with a clear predominance of good health, the objective health distribution showed a completely divergent pattern, with most participants displaying poor or intermediate health and only 4.2 % displaying good health. Another manifestation of this disparity was the high prevalence of overrating, *i.e.*, 101 cases of clear overrating (18 % of the population), and 130 plus 174 cases of moderate overrating, amounting to 73 % of the population with some degree of overrating (Table 2). Of the 265 residents who received good physician ratings, only 9.2 % had good objective health, and 39.0 % had poor objective health (Table 2). Agreement between self- and physician-rated health, between objective and physician-rated health, and between objective and self-rated health were all moderate to poor (weighted kappas of 0.23, 0.23 and 0.12, respectively).

**Table 1:** Selected baseline characteristics of residents living in facilities for older people in Madrid, Spain (1998/9)

Variables	No. (%)^a^
Total	740 (100)
Age group, years	
65–74	98 (12.3)
75–84	308 (40.8)
≥85	334 (46.9)
Sex	
Women	408 (75.4)
Men	332 (24.6)
Type of facility	
Public	405 (44.4)
Subsidized	78 (8.4)
Private	257 (47.2)
No. of medical conditions	
0–1	156 (20.8)
2–3	308 (42.2)
≥4	276 (37.1)
Functional dependency^b^	
Independent (100)	192 (21.9)
Mild/ Moderate (61–99)	333 (47.3)
Severe/Total (0–60)	198 (30.8)
Missing	17
Cognitive impairment	
Non-impaired	317 (50.8)
Mild/Moderate impairment	179 (32.2)
Severe impairment	84 (17.0)
Missing	160
Self-rated health	
Very good/good	355 (54.9)
Fair	208 (30.1)
Poor/very poor	98 (15.0)
Missing	79
Physician-rated health	
Very good/good	349 (50.6)
Fair	234 (36.3)
Poor	77 (13.0)
Missing	80
Objective health	
Good	40 (4.7)
Intermediate	210 (35.0)
Poor	380 (60.3)
Missing	110

^a^Unweighted counts and weighted percentages

^b^Barthel Index score

**Table 2:** Objective health and physician-rated health distributions

	Physician-rated health			
Objective health	Very good/good	Fair	Poor/very poor	Total
	No. (%) ^a^	No. (%) ^a^	No. (%) ^a^	No. (%) ^a^
Good	34 (9.2)	1 (0.2)	0 (0.0)	35 (4.3)
Intermediate	130 (51.8) ^b^	44 (20.5)	6 (7.5)	180 (32.9)
Poor	101 (39.0) ^c^	174 (79.3) ^b^	69 (92.5)	344 (62.8)
Total	265 (100)	219 (100)	75 (100)	559 (100)

^a^ Unweighted counts and weighted percentages

^b^ Moderate overrating

^c^ Clear overrating

Table 3 shows the physician-rating behavior pattern by reference to the components used to build the objective health scale, in order to ascertain the potentially different importance attached to these components. It will be noted that functional dependency and number of conditions showed clearly more weight in physician rating than did cognition. For each increase of 1 in the level of severity of functional dependence, the (adjusted) probability of receiving a poor physician rating was nearly 6 times higher than that of receiving a good rating (relative risk ratio (RRR): 5.94). In changing from good to fair, the number of conditions had slightly more influence than did functional dependence (RRR, 3.05 vs. RRR, 2.05), whereas in changing from fair to poor, functional dependence had clear predominance (RRR, 3.11 vs. RRR, 5.94).

**Table 3:** Association between components of objective health and physician-rated health

	Physician rating		
Variable ^a^	Good	Fair	Poor
	RRR ^b^ (95%CI)	RRR ^b^ (95%CI)	RRR ^b^ (95%CI)
Functional dependency	1.00 (reference)	2.05 (1.35-3.11)	5.94 (2.77-12.77)
Medical conditions	1.00 (reference)	3.05 (2.20-4.21)	3.11 (1.87-5.17)
Cognitive status	1.00 (reference)	1.37 (0.89-2.13)	1.47 (0.76-2.83)

^a^ Entered in models as 1, 2 or 3, for the low, intermediate and high severity level, respectively

^b^ Relative risk ratios of receiving fair and poor, as compared to good, physician rating for each increase of 1 in the level of severity of the independent variables, adjusted for baseline age, sex, facility type and the 3 components of the table, from a multinomial logistic regression model

### Mortality

**Fig. 2:**
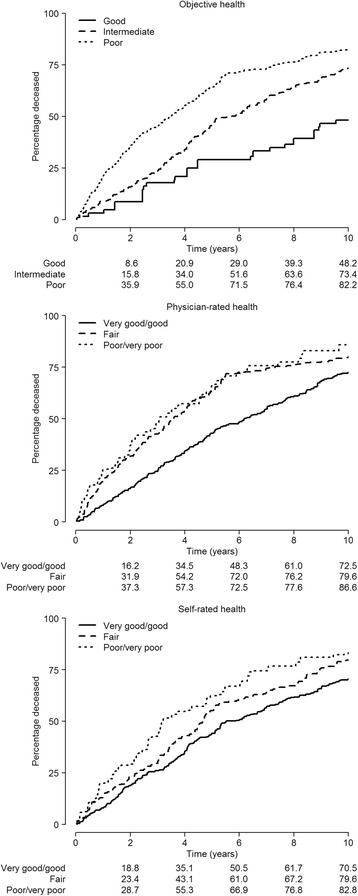
Non-parametric survival curves by objective health, physician-rated health, and self-rated health strata among nursing-home residents in Madrid, Spain, 1998–1999 through 2013, obtained from the baseline survival functions of health-stratified Cox models (good, fair, or poor health) with years from the baseline interview as the time scale. Models were adjusted for age, sex and type of facility

Of the 754 participants at baseline, 598 died and 101 were deemed to be alive; since there was insufficient information on the remaining 55, they were deemed to be missing and dropped from the survival analysis. Median and maximum follow-up time was 4.49 and 15.25 years, respectively. The unweighted mortality rate was 144.6 per 1000 person-years (598 deaths during the 4135 person-year follow-up). Table 4 shows the hazard ratios for the main variables. Mortality was clearly associated with functional dependency, cognitive impairment and the three variables used to assess health, *i.e.*, self-rated health, physician-rated health, and objective health. With the exception of physician-rated health, estimates behaved in a graded fashion, in line with what would be expected. Physician-rated health estimates were the same for health assessed as fair and poor. All these results can be better appreciated in Fig. 2, which shows the fine, graded behavior of the objective health measure, and the somewhat unexpected “dichotomization” of physician rating, showing no difference in the probabilities of death for persons rated as having “fair” or “poor” health, regardless of the duration of follow-up. Table 5 shows the results of these three health estimates when mutually adjusted (in the same Cox model). Physician-rated health lost its previous association with long-term mortality, while objective health weakened and self-rated health maintained a clear, graded association. It should be mentioned that this last model included only residents with non-missing values of self-rated health, thus constituting a subgroup which excluded most subjects with severe cognitive impairment.

**Table 4:** Hazard ratios for mortality, by baseline sociodemographic characteristics and health conditions of those residents with valid mortality data

Baseline variable	No. of subjects ^a^ (%)	No. of person-years	No. of deaths	Hazard ratio ^b^ (95 % CI)
Overall	699 (100)	4,134.6	598	
Age (years)				
65–74	95 (12.7)	774.9	68	1.00 (reference)
75–84	287 (39.9)	1,904.7	248	1.48 (1.11–1.99)
≥85	317 (47.4)	1,455.0	282	2.19 (1.52–3.16)
Sex				
Women	386 (75.7)	2,343.0	331	1.00 (reference)
Men	313 (24.3)	1,791.6	267	1.11 (0.94–1.31)
Type of facility				
Public	401 (47.0)	2,348.4	357	1.00 (reference)
Subsidized	72 (8.0)	369.3	60	1.14 (0.90–1.44)
Private	226 (45.0)	1,416.8	181	0.84 (0.66–1.08)
No. of chronic conditions				
0–1	151 (21.6)	1,038.6	125	1.00 (reference)
2–3	290 (42.0)	1,851.0	245	0.99 (0.77–1.26)
≥4	258 (36.4)	1,245.0	228	1.21 (0.96–1.54)
Functional dependency				
Independent	187 (22.1)	1,465.7	154	1.00 (reference)
Mild/moderate	316 (47.0)	1,910.9	270	1.37 (1.14–1.63)
Severe/total	179 (28.4)	688.9	157	2.27 (1.78–2.89)
Unknown	17 (2.5)			
Cognitive impairment				
Unimpaired	297 (41.6)	2,069.6	246	1.00 (reference)
Mild/moderate	169 (26.8)	798.1	145	1.38 (1.05–1.81)
Severe	79 (14.0)	283.1	70	2.11 (1.45–3.05)
Unknown	154 (17.6)			
Self-rated health				
Very good/good	336 (48.1)	2,309.3	274	1.00 (reference)
Fair	197 (26.4)	1,137.0	174	1.28 (1.07–1.53)
Poor/very poor	95 (13.5)	443.8	85	1.58 (1.17–2.12)
Unknown	71 (12.0)			
Physician-rated health				
Very good/good	329 (42.7)	2,291.5	275	1.00 (reference)
Fair	222 (31.1)	1,059.7	196	1.41 (1.15–1.73)
Poor/very poor	69 (10.3)	289.6	61	1.55 (1.02–2.35)
Unknown	79 (15.9)			
Objective health ^c^				
Good	40 (4.4)	367.3	29	1.00 (reference)
Intermediate	199 (31.2)	1,376.1	161	1.27 (0.84–1.91)
Poor	351 (51.6)	1,631.5	310	1.83 (1.27–2.63)
Unknown	109 (12.8)			

^a^ Unweighted sample counts and weighted percentages based on the underlying population distribution

^b^ Hazard ratios and 95 % confidence intervals (CIs) were obtained from Cox models, with years from the baseline interview as the time scale, adjusted for baseline age, sex, and type of facility, taking into account the stratified cluster sampling and the different selection probabilities

^c^ Residents presenting with 0–1 chronic conditions, functionally independent in basic activities of daily living, and having unimpaired cognition were assigned to good objective health; those presenting with ≥ 4 chronic conditions, severe/total functional dependency, or severe cognitive impairment were assigned to poor objective health; and the remaining residents presenting with intermediate severity levels were assigned to fair objective health

**Table 5:** Hazard ratios for mortality, by physician-rated health, objective health and self-rated health

	Health estimate ^a^		
Level	Physician-rated health	Objective health	Self-rated health
Good	1.00 (reference)	1.00 (reference)	1.00 (reference)
Intermediate	1.13 (0.88–1.46)	1.19 (0.89–1.58)	1.29 (1.02–1.64)
Poor	1.06 (0.58–1.92)	1.32 (0.97–1.81)	1.51 (1.05–2.18)
*P* for linear trend	0.63	0.25	0.01

^a^ Hazard ratios and 95 % confidence intervals obtained from a proportional hazards model, with years from the baseline interview as the time scale, adjusted for baseline age, sex, type of facility, physician-rated health, objective health, and self-rated health, taking into account the stratified cluster sampling and the different selection probabilities

## Discussion

To our knowledge, this is the first time that physician-rated health has been assessed against an objective measure. There was a notable degree of discordance, and, in particular, a high fraction of clear overrating, inasmuch as near four out of ten of those rated by a physician as having good (or very good) health, were shown to have poor objective health (Table 2), a proportion corresponding to 18 % of the entire population; and although the literature provides no benchmark against which this can be compared, it strikes us as being a high fraction. Population distribution of physician- and self-rated health were very similar, with a slightly worse assessment by doctors, yet both were far better than the ad hoc measure of objective health used (Fig. 1). It would appear that both residents and physicians undervalue the health aspects used to construct the objective measure, *i.e.*, functional dependency, multimorbidity, and cognitive impairment. Individuals are known to contextualize [1], and this phenomenon can be very pronounced in a nursing home setting. It is worth mentioning that self-rated health was better among Madrid’s institutionalized population than its community-dwelling counterpart [15, 16]. While some degree of contextualization by residents –and even by physicians– might be considered reasonable, what we found was nevertheless excessive, with consequences that remain to be studied. Some physicians may regard multiple chronic conditions, disability or cognitive impairment as normal or as being in a steadily progressive state, possibly with no likelihood of improvement, and so opt for a favorable rating in cases having no other complications, confining their poor evaluations to cases having poor prognoses (*e.g.*, short life expectancy). Then again there are times when physicians might be very familiar with their patient’s status and reckon that, despite the objective facts, their patient feels well. Having said this, however, there might well be instances where insufficient knowledge of the patient is also a real possibility. We did not find comparable works but a related approach is worth mentioning. Kroenke *et al.* studied how well physicians’ global estimates of disease severity (for patients with chronic cardiac or pulmonary disease) corresponded to more specific prognostic variables assessed by them in the same 6-item questionnaire, in an attempt to check physicians’ internal consistency [17]. They found a strong association between the global assessment and each of the specific elements. On the other hand physicians’ and patients’ global estimates were weakly correlated, suggesting that physicians and patients may weight different aspects of disease severity. In our study agreement between self- and physician-rated health, between objective and physician-rated health, and between objective and self-rated health were all moderate to poor (weighted kappas of 0.23, 0.23 and 0.12, respectively).

Analysis of *mortality* indicated a good, graded pattern of behavior by the objective health measure, and an unexpected pattern of behavior by physician-rated health. It seems that physicians effectively use only two evaluations, namely, “good” or “not good”. Examination of mortality associated with physician ratings, with additional adjustment for important variables such as functional dependence, cognition and number of chronic conditions, showed a clearer association with the “fair” (HR: 1.15 (95 % CI 0.91-1.46)) than with the “poor” category (HR: 1.08 (95 % CI 0.61-1.92)). It should perhaps be recalled here that the latter category included “poor” and “very poor”. Other studies have focused on the predictive ability of physician ratings: Giltay *et al.* reported a better prognostic value for self-rated health than for physician-rated health when it came to predicting cancer mortality, while the opposite was true when it came to predicting cardiovascular deaths [5]; and a study on community-dwelling Taiwanese elders by Todd and Goldman yielded unexpected findings, in that physician ratings were observed to have a weak predictive power, and a clear, weaker predictive capacity as compared to both self-rated and interviewer-rated health [6].

### Objective health construct

No similar measure was to be found in the literature. Our aim was to incorporate important, undisputed elements of health status, while excluding any subjective judgment. It is our considered opinion that the dimensions used (functioning, cognition and chronic morbidity) are essential in any health construct but this is open to discussion [18]. Although the proposed objective health measure ought to be tested, we nonetheless believe that it has a role in highlighting the main problem detected in our population, *i.e.*, overrating. We observed a higher-than-expected number of residents with unquestionably poor objective health who received a good rating from a physician. By “unquestionably”, we wish to convey that this particular aspect of the measure does not necessarily require a formal validation process because, in our opinion, anyone suffering from a severe degree of cognitive impairment, a severe degree of disability in basic activities of daily living, or ≥4 chronic conditions should not and cannot be considered to enjoy good health by any health professional conversant with the meaning of these components. We thus feel that it could be a good measure for evaluating health-overrating phenomena.

Our measure would have some valuable advantages. Equivalent measures can be easily constructed by anyone with available data on any accepted measure of ADL dependency (*e.g.*, Katz’s ADL scale [19]) and cognitive impairment (such as the Mini-mental State Examination [20]), and a sensible list of chronic conditions, thus facilitating reliable comparability across populations. In addition, it focuses on a stable chronic state rather than episodic poor health. Needless to say, all these issues ought to be adequately tested in various settings and in different populations. In addition the association with mortality, which was extremely clear and graded (see Fig. 2 and Table 4), confers validity based on unquestionably objective standards.

### Limitations

First, systematic information was not collected on relevant physician characteristics, such as personal attributes, medical specialty, work load, or frequency of visits. The majority of the medical practitioners interviewed were primary care or family physicians.

As with most measures, our objective health construct is liable to error. For instance, some residents may be misclassified and placed in the severe category of any of the three component variables constituting the measure, and so be wrongly assigned to poor objective health. Even in such a case, however, overall discordance will still be very notable because there are also many instances of “moderate” discordance (good physician rating and intermediate objective score, or fair physician rating and poor objective score). Furthermore, there is the possibility of some degree of subjectivity when physicians answer the questionnaire, *e.g.*, transient ischemic attacks might be regarded as cerebrovascular disease by some physicians but not by others, and the same could occur in the case of mild hypertension. These add to the sources of misclassification in this study, which we do not see as being important in epidemiologic terms. Finally, the National Death Index might have missed some deaths that were not confirmed. Nonetheless, the random nature of these potentially missing deaths could be expected to have a minimal impact on most estimates.

## Conclusions

There were notable discrepancies between physician-rated and objective health, particularly in the form of overrating, which may possibly have corresponded to an excessive contextualization process, with consequences that remain to be studied.

Physician-rated health may be very valuable and is, in general, easy to obtain, but evidence of its real usefulness can only be furnished by further research into its properties. Although this study has highlighted a negative aspect of physician-rated health, adequate design and selected information may well serve to better understand it. In addition, other settings may possibly reveal the opposite manifestation of discordance, *i.e.*, underrating, along with its potential consequences of overdiagnosis, overtreatment, and related facts.

Much of the speculation regarding the reasons for discordance could be elucidated by research which targeted physicians, questioned them more closely and requested that they give an explanation for their ratings. Future research should collect data that focus on physician and facility characteristics. Research conducted in other settings would also be useful.

## Abbreviations

ADL: Activities of daily living
MDS-COGS: Minimum data set cognition scale
RRR: Relative risk ratio
SPMSQ: Short portable mental status questionnaire
